# Experimental and Numerical Study on the Failure Characteristics of Brittle Solids with a Circular Hole and Internal Cracks

**DOI:** 10.3390/ma15041406

**Published:** 2022-02-14

**Authors:** Chengjun Le, Xuhua Ren, Haijun Wang, Shuyang Yu

**Affiliations:** 1 College of Water Conservancy and Hydropower Engineering, Hohai University, Nanjing 210098, China; lecj@hhu.edu.cn (C.L.); renxh@hhu.edu.cn (X.R.); yushuyang_hhu@163.com (S.Y.); 2 Sichuan Woneng Investment Group Co., Ltd. Chengdu 610000, China; 3 Nanjing Hydraulic Research Institute, Nanjing 210098, China

**Keywords:** 3D-ILC, fracture mechanics, crack propagation, problem with circular hole, brittle solids, 3D internal crack

## Abstract

A stress analysis of a circular hole is one of the classical problems in mechanics. Internal cracks are inherent properties of materials, and they are mostly three-dimensional in form. However, studies on hole problems with three-dimensional internal cracks are still lacking. In this paper, internal cracks were generated in brittle materials containing circular holes based on 3D internal laser-engraved crack technology. Then, uniaxial compression tests were performed. The experimental results were compared with the existing literature, and theoretical and numerical simulation studies were carried out. The results show that: (1) The main crack shapes are the primary cracks and remote cracks. (2) The dynamic fracture characteristics existed in the formation of primary cracks and the surface of remote cracks. The tips of primary cracks were arc-shaped, and the surfaces of the remote cracks were curved. Remote cracks were tangential to the orifice where type III spear-like characteristics appeared. (3) The stress birefringence technology can be combined with 3D internal laser-engraved crack technology for internal crack stress information monitoring, the moire around the orifice was “flamboyant”, and the moire at the tip of the prefabricated crack was “petallike”. (4) The existence of internal cracks reduced the cracking and breaking load of the specimen, and compared with the intact orifice specimen, the upper primary crack, the lower primary crack, the remote crack and the failure load were reduced by 41.2%, 31.7%, 15.9%, and 32.3%, respectively. (5) The results of qualitative stress analysis of the orifice specimen were consistent with the initiation law of primary cracks and remote cracks. The K distribution based on M integral and the numerical simulation of crack propagation process based on the maximum tensile stress criterion were consistent with the law of primary crack growth. Compared with the current mainstream method of transparent rock research, 3D internal laser-engraved crack technology has certain advantages in terms of brittleness, crack authenticity, stress field visualization, and fracture characteristics, and the result will provide experimental and theoretical references for research on three-dimensional problems and internal cracks in fracture mechanics.

## 1. Introduction

The stress and failure analysis of circular holes is one of the classical problems in the mechanics, for example, the orifice fatigue fracture problems in aviation field [1,2,3]; the stress concentration problems of orifice bolts in mechanical engineering [4,5,6]; the problems of perforating hydraulic fracturing in oil and shale gas extraction [7,8,9]; the stability of surrounding rock of tunnels in hydraulic and geotechnical fields [10,11,12]. Research on the stress and failure analysis of circular holes mainly concentrates on three aspects: (1) Theoretical research. For example, Lamé et al. [13] gave a theoretical solution of a ring subjected to uniform internal and external loads based on the linear elasticity theory, which later became the basis of inelastic orifice problems; Muskhelishvili et al. [14] used the plane elastic complex function method to give the expression for the stress field and displacement field of the orifice problem; Lv et al. [15] and Fan et al. [16] obtained the analytical expression of an orifice with arbitrary shape by deriving the orifice mapping functions; Mendelson et al. [17] extended the elasticity problem of planar orifices to elastoplasticity. (2) Experimental studies, for example, Zhang et al. [18] conducted experimental studies on mechanical properties and failure models of marble under different numbers and diameters of orifices; Gong et al. [19] analyzed the failure processes of granite specimens with rectangular holes under triaxial stress conditions; Zhu et al. [20] discussed the failure processes of sandstone with double round holes under uniaxial compression. (3) Numerical simulation, for example, Li et al. [21] used Flac3d software to simulate the stress, strain, and damage process of plate samples with bilateral orifice; Li et al. [22] used particle flow discrete element software PFC to conduct numerical simulation research on the uniaxial compression failure process of orifices containing circular, rectangular, and gate shapes; Xie et al. [23] used the damage and fracture software RFPA-3D to simulate the three-dimensional fracture of cuboid samples with orifices. 

With the continuous advances in fracture mechanics research, the mechanisms of interactions of pre-existing cracks and orifice have also been further studied. For example, Wu et al. [24] conducted experimental and numerical simulation studies on cement mortar samples with circular holes and cracks; Zhu et al. [25] discussed the strength characteristics, deformation characteristics, and fracture evolution processes of tabular sandstone with prefabricated orifice and fissures; Zhang et al. [26] made an in-depth analysis of the mechanisms of rock mass with orifice and fracture after grouting. However, most of the previous studies on cracks are limited to two dimensions, and the research on the orifice with 3D crack is quite rare. In fact, simply transforming 3D problems into 2D cannot fully reflect the mechanical properties of defective materials [27]. The reasons contributing to the lack of research on 3D internal cracks are mainly reflected in the following aspects: (1) Internal crack visualization problems, for example, Li et al. [28] used the cement mortar pouring method to bury three-dimensional cracks, but they could not observe the propagation process of three-dimensional cracks during the test process. Although CT scanning [29] and acoustic emission technology [30] have been introduced, they still cannot solve the problems of scanning accuracy, accurate positioning, and real-time observation. (2) The generation of internal cracks, for example, with the continuous development of research, transparent brittle materials have been developed to facilitate the observation of internal cracks. There are three main methods to generate internal cracks in transparent brittle materials: (1) The cut and paste method [31]. This method was firstly proposed by Adams [31] in 1978. This is carried out by cutting the semi-circular surface crack into two pieces of plexiglass and forming an “inner crack” by almosaics, which obviously destroys the integrity of the original test when using this method. (2) Embedded casting method [32]. This method was firstly proposed by Dyskin [32] in 1994. It is formed by pouring epoxy resin into a metal or mica sheet that is prepositioned in a mold (regarded to be an internal crack). This method solves the problem of sample integrity of the cutting and pasting method and has been regarded as a mainstream research method for three-dimensional internal crack by scholars all over the world. However, this method requires casting molding samples at very low temperature (minus 20~30 degrees) to maintain brittleness, and the highest brittleness can only reach 1/3~1/7. At the same time, the test discreteness under low temperature is very large, the test success rate is low. In addition, regarding the heterogeneous sheet as an internal crack is not consistent with the actual situation, and it is difficult to imagine that the crack surface is a hard metal or mica sheet. (3) 3D printing method. This method is considered to be the most promising new technique for solving 3D internal crack generation problems. The advantage lies in that it can be modeled digitally and can produce arbitrary internal cracks. For example, Jv et al. [33] used 3D printing to prepare coal and rock models with complex fracture networks; Liu et al. [34] prepared transparent rocks using 3D printing. However, 3D printing technology still has many problems to be solved: firstly, the method still uses resin material for printing, which cannot guarantee enough brittleness; secondly, 3D printing internal cracks still needs to use heterogeneous support materials, essentially similar to the above embedded pouring method.

Therefore, this paper adopted a series of methods to solve the above problems. First, 3D-ILC technology proposed by the author [35] was used to generate the 3D internal cracks in a brittle solid without any heterogeneous support materials and, at the same time, did not have any impact on the surface of the brittle solid; The transparent brittle material glass was selected to solve the problems of observing the process of crack propagation. At the same time, the brittleness was higher, which provided a reference for similar research on natural brittle materials such as rock. The stress birefringence of glass lays a foundation for stress visualization. Based on this, uniaxial compression tests were carried out on the specimens with different embedded depths of internal cracks. The fracture mechanism and mechanical characteristics of the specimens with three-dimensional internal cracks were revealed through stress moire, failure pattern analysis, and numerical simulation. The results provide a physical test basis for the corresponding theoretical research and correction.

## 2. Research Scheme and Sample Preparation

### 2.1. 3D-ILC

3D-ILC was proposed by Wang et al. [35] in 2017, which realized the fabrication of arbitrary internal cracks in a material without any impact on the surface and be called the 3D internal laser-engraved crack technology.

It is difficult to make a purely closed internal crack. It can be imagined that it is impossible to make a “surgically” precise structural change in the interior of a complete object without affecting other parts. However, electromagnetic and wave physical fields can penetrate objects and be used in the interior. Wang et al. [35] finally proposed that 3D-ILC acts on the interior of materials through electromagnetic fields to form plasma (the fourth state of matter) blasting, thus producing macroscopic pure closed internal cracks. Due to the penetration of electromagnetic field, the internal crack can be made without any influence on the surface by controlling the parameters.

### 2.2. Sample Material

The glass was selected for testing due to the following advantages: (1) due to the homogeneity and isotropy, glass strictly follows Hooke’s law before crack propagation, and is a classic material in brittle solid fracture mechanics; (2) glass is a stress birefringent photosensitive material, which provides a basis for direct observation of stress distribution; (3) as an index to measure the brittleness of materials, the pull-to-pressure ratio of glass can reach 1/13~1/33, which is similar to that of rock materials (1/12~1/35), and can better simulate the brittleness of rock.

As is pointed out by Li [36] in his book, *All Pioneering Tests of Solid Mechanics Use Glass as the Basic Sample Material, and Fracture Mechanics is No Exception*, the Griffith fracture criterion, the basis of modern fracture mechanics, uses glass as a test material. Subsequently, many scholars have carried out classical tests based on glass. For example, Roesler et al. [37] conducted three-dimensional Hertz contact fracture tests on glass materials and observed conical crack morphology; Knauss et al. [38] studied the morphology of pure type Ⅲ crack with glass material and observed the “quasi-spiral” failure characteristics; Sommer et al. [39] used glass rods to conduct tensile and torsion tests and observed the classic fracture form of I–III “double spearhead”, which became the permanent cover of the international journal, *Engineering Fracture Mechanics*. 

### 2.3. Experimental Conditions

For comparison, two types of brittle solid samples were used to carry out the tests: (1) intact samples (group A); (2) samples with internal crack (group B and group C), and the design, number, test purpose, and research content of each sample are shown in Table 1.

(1) Group A (complete orifice sample without internal cracks). The control group with no internal cracks, the samples were loaded to crushing to provide comparison for the orifice samples (Group B and Group C) with internal cracks of different buried depths, which is marked as A0.
(2) Group B (orifice samples with internal cracks of different buried depths). Samples buried at depths (*d*) 0.5, 1, 1.5, and 2 cm, which are marked B0.5–B2.5. The samples were loaded to crushing to observe the propagation of internal crack, the characteristic loads and other information during the loading process, and the fracture morphology was observed after the specimen was crushed.
(3) Group C. The samples were the same as group A and B, but the loading stopped when the main crack and the remote crack appeared, which were recorded accordingly. The samples were marked Ci-j, where i ranges from 0 to 2, representing samples without internal cracks and samples with cracks in different buried depths. The values of j range from 1 to 2, representing the occurrence of main cracks and remote cracks.

### 2.4. Sample Preparations

**Figure 1:**
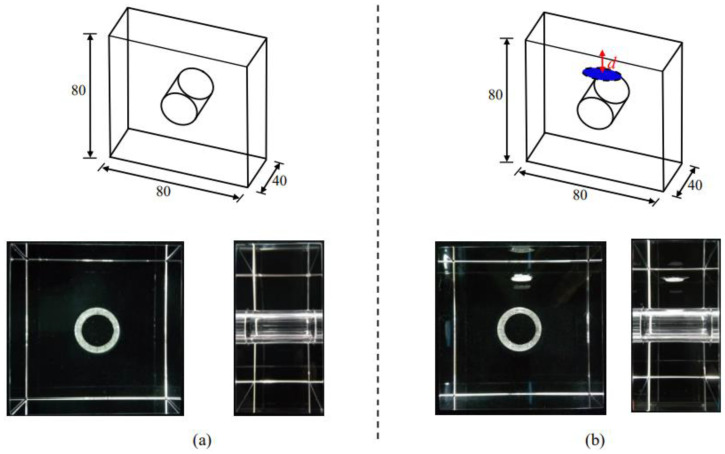
Sample design: (**a**) samples without internal cracks; (**b**) samples with internal cracks.

(1) Group A (samples without internal cracks): The sample was a cubic sample of 80 × 80 × 40 mm^3^ with a circular hole in the center, with a diameter that was 20 mm as shown in Figure 1a.
(2) Group B and C (samples with internal cracks): The sample was a cubic sample of 80 ×80 × 40 mm^3^, with a circular hole in the center and a circular inner crack, with a diameter that was 2c = 13 mm. The *d* between the inner crack and the top surface of the sample was 0.5, 1, 1.5, and 2 cm, respectively, as shown in Figure 1b.

### 2.5. Test Instruments

**Figure 2:**
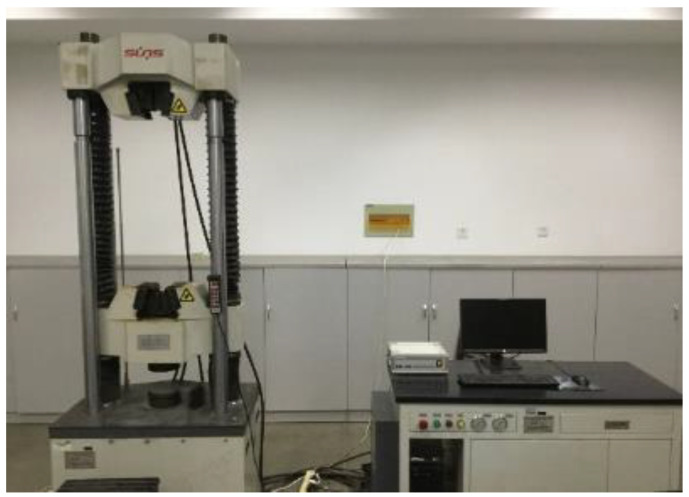
Loading system.

**Figure 3:**
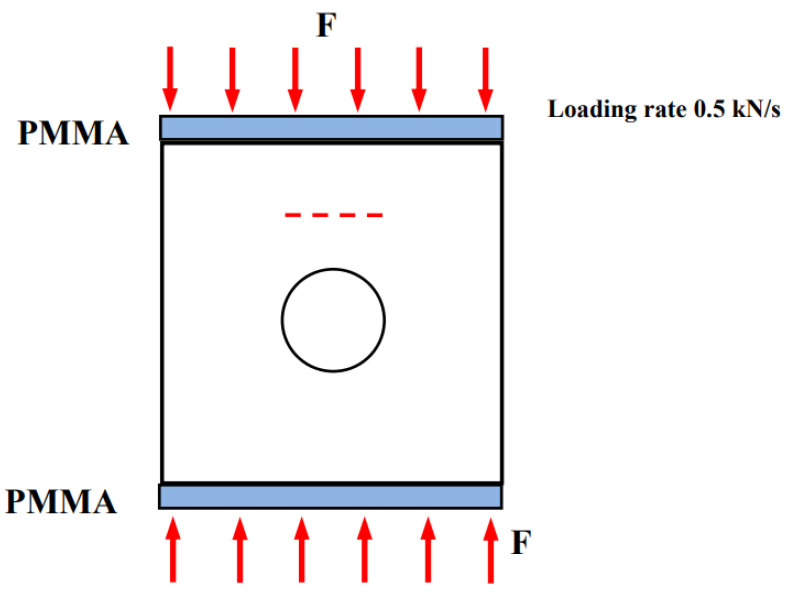
Uniaxial compression loading mode.

The SUNS-650W loading equipment (Tinius kuli, Suzhou, China) was applied in this experiment, which had two loading modes. The maximum loading pressure was 600 kN, and the displacement range was 0–200 mm. In our experiment, the force control loading mode was utilized, and the loading rate was 0.5 kN/s. The equipment is shown in Figure 2, and the loading mode is shown in Figure 3. Stress concentration is more sensitive in brittleness test, and the unevenness or unsmoothness of the end will lead to cracks formed by stress concentration at the end of the sample, which will affect the test quality. To avoid this and reduce the end friction effect, smooth PMMA(plexiglass) is placed at the top and bottom.

### 2.6. Birefringence Effect Observation System

Glass and other amorphous media usually exhibit optical isotropy in a stress-free state. When subjected to stress, the refractive index characteristics change, showing optical anisotropy. When a beam of light passes through glass with internal stress, it will produce two beams of light with different propagation speed, ordinary light *o* following the law of refraction, and extraordinary light *e* not following the law of refraction, which is stress birefringence. According to the stress-optical law, when the incident light incident on the test object due to the birefringence effect, the relationship between the principal stress and the corresponding refractive index is as follows:

*n*_1_ − *n*_2_ = (*C*_1_ − *C*_2_)(*σ*_1_ − *σ*_2_) (1)

where *σ*_1_ and *σ*_2_ are the principal stresses of the sample under uniaxial compression stress state, respectively; *n*_1_ and *n*_2_ are the refractive indices of the direction of *σ*_1_ and *σ*_2_, respectively; *C*_1_ and *C*_2_ are the material stress-optical coefficients. The optical path difference (Δ) generated by polarized light passing through the sample is:

Δ = (*n*_1_ − *n*_2_)*h* (2)

where *h* is the thickness of the medium. According to Equations (1) and (2), the corresponding relationship between stress and optical quantity is established:

(*σ*_1_ − *σ*_2_) = Δ/*hC* (3)

**Figure 4:**
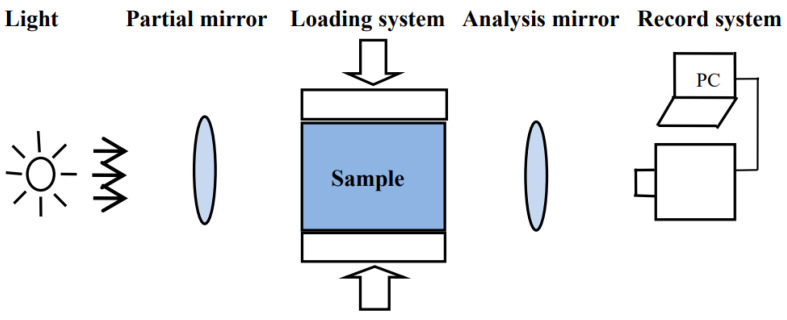
Arrangement of stress birefringence effect test.

The arrangement of orthogonal plane-polarized light field is shown in Figure 4.

## 3. Experimental Results

### 3.1. Failure Process Analysis

#### 3.1.1. Orifice Sample without Internal Cracks (Group A)

**Figure 5:**
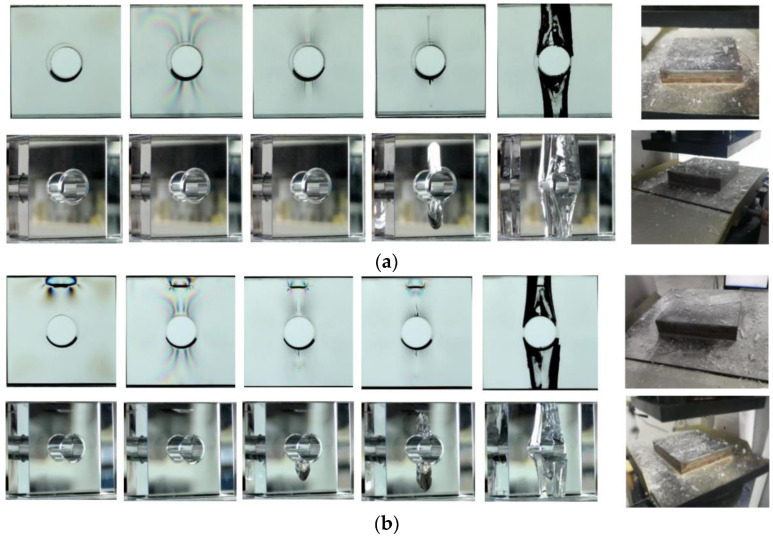

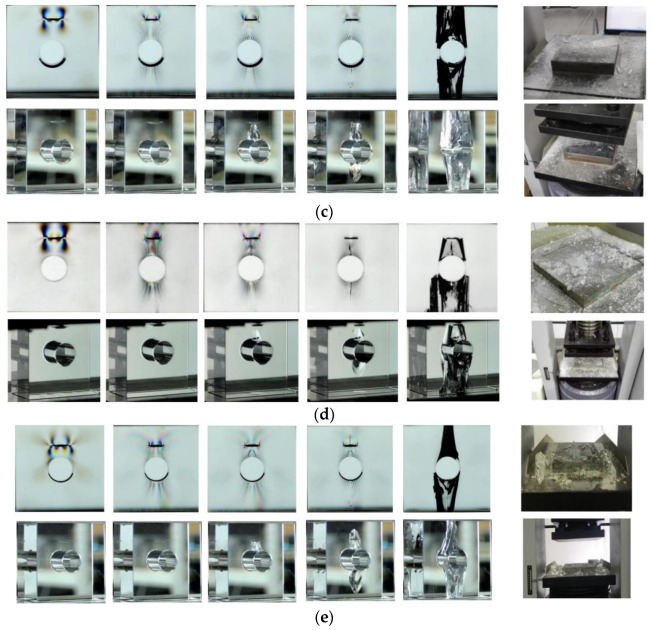
Crack propagation processes of the orifice specimen. (**a**) A0; (**b**) B0.5; (**c**) B1; (**d**) B1.5; (**e**) B2.

As can be seen from Figure 5a, the typical failure process of the sample under uniaxial compression can be divided into four stages: (1) stable stage; (2) main crack propagation stage; (3) remote crack propagation stage; (4) failure stage.

(1) Stable stage: This stage persists until the loading force to 131.16 kN. No crack propagation was observed in this stage;
(2) Main crack propagation stage: when loading to 131.16 kN, the upper and lower main cracks initiated almost simultaneously, and the main crack’s growth rate gradually decreased after the crack initiation.

**Figure 6:**
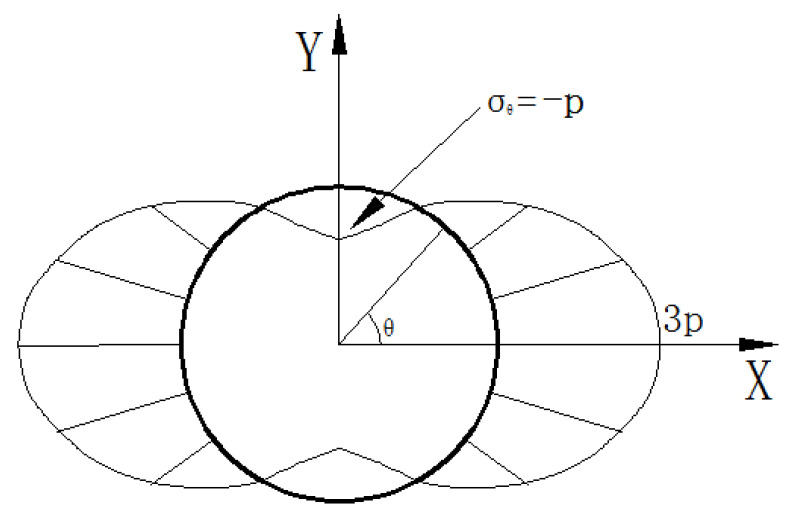
Stress distribution around the orifice.

The glass can be regarded as a completely elastic body before cracking. According to The Ramet solution (Equation (4)), the annular stress distribution at the orifice is similar to a “peanut shell”, which is shown in Figure 6; that is, the maximum annular tensile stress concentration occurs at the top of the orifice. Therefore, under the action of the maximum annular tensile stress, a mode I tensile crack first appears above and below the orifice, and the direction is perpendicular to the annular tensile stress.

(4) $$\{\begin{array}{c} \sigma_{r}=0\\ \sigma_{\theta }=p+2\mathrm{pcos}2\theta \\ \tau_{r\theta }=0 \end{array}$$

It is noteworthy that the crack tip is circular in the main crack propagation process. Assume that the length and length radius of the crack tip are ellipses of a and c, respectively, the vertical stress is *σ*_A_, and the following equations can be obtained:

(5) $$K_{Ι}=\varphi \sigma_{A}c^{0.5}$$

(6) $$\varphi \;(\frac{a}{c},\;\beta )=\pi^{0.5}{\left[\cos^{2}\beta +{\left(\frac{c}{a}\right)}^{2}\sin^{2}\beta \right]}^{0.5}/\;E\;(\frac{a}{c})$$

(7) $$E\;(\frac{a}{c})={\int_{0}^{\frac{\pi }{2}}\left[1-(1-\frac{c^{2}}{a^{2}})\;\sin^{2}\phi \right]}^{0.5}d\phi$$

where K_Ι_ is the mode I stress intensity factor; c is the crack length; *φ* is the geometry item; E(a/c) is the elliptic integral; *ϕ* is the dummy variable. When a/c ≠ 1, then:

(8) $$K_{Ι}(\beta =\frac{\pi }{2})/K_{Ι}(\beta =0)={\left(\frac{c}{a}\right)}^{0.5}\leq 1$$

Therefore, without external interference, crack propagation always tends to form a circular tip with a/c = 1.

(3) Remote crack propagation stage: The orifice sample with no internal cracks did not continue to exhibit “bisection” fracture failure along the main crack, but it produced two pairs of remote cracks at the symmetrical positions above and below the orifice when the load was 438.25 kN. The surface was curved and tangent to the orifice. The line between the tangent point and the center of the circular hole was at an angle of approximately 30° with the horizontal axis. The occurrence time of the upper and lower remote cracks is similar;
(4) Failure stage: When the failure load reached 587.13 kN, the sample showed violent “explosive” failure, and the sample was detrital with a long range of avalanche. This characteristic is typical of brittle material failure under ballast loads of a rigid testing machine.

#### 3.1.2. Orifice Sample with Internal Cracks (Group B)

As can be seen from Figure 5b–d, the typical failure process of the sample with three-dimensional internal cracks under uniaxial compression was similar to that of the complete sample without cracks, which can be divided into four stages: (1) stable stage; (2) main crack propagation stage; (3) remote crack propagation stage; (4) failure stage.

(1) Stable stage: This stage persisted until a loading force of 77.12 kN. No crack propagation was observed during this stage.
(2) Main crack propagation stage: When loading to 77.12 kN, the upper main crack first initiated, and when the load reached 89.61 kN, the lower main crack initiated. The main crack initiation load of samples containing internal cracks was less than that of samples not containing internal cracks. Meanwhile, the internal cracks also influenced the stress field of the sample, which led to the upper main crack becoming easier to initiate than the lower main crack. What should be noticed is that for the samples with lower buried depths (*d* = 0.5, 1, and 1.5 cm), the main crack would not “penetrate” the prefabricated internal crack. However, when the buried depth is higher (*d* = 2 cm), the main crack would “penetrate” the prefabricated internal crack;
(3) Remote crack propagation stage: When loaded to 368.72 kN, remote cracks appeared, and the crack form was similar to that of the sample without internal cracks, but the initiation load of remote cracks was smaller than that of the sample without internal cracks;
(4) Failure stage: When the load reached 397.61 kN, there was a violent “explosive” failure, and the failure load was significantly lower than that of the complete orifice sample without crack.

### 3.2. Stress Moire Laws

**Figure 7:**
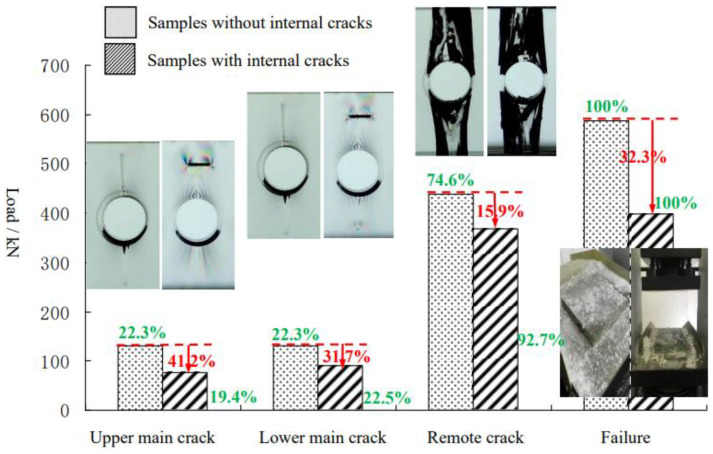
Characteristic load distributions.

The changes of stress moire in the test during the loading process is shown in Figure 7, which mainly shows the following rules:

(1) When the pressure was relatively lower, the stress moire presented the shape of a “flame” in the top and bottom of the orifice for the condition of the sample without internal cracks. However, for the condition of the sample with internal cracks, the “petal shape” stress moire was observed in the tips of internal crack;
(2) With the increase in load, the petal moire at the tip of the prefabricated crack gradually became lighter, while the flame moire at the upper and lower part of the orifice gradually highlighted;
(3) The appearance of the main crack in the upper and lower part of the orifice changed the moire distribution of the “flame shape” and made the moire appear similar to an “inverted triangle” along the direction of the main crack propagation.

Due to the difficulty of observing internal cracks, visualization of the stress field inside rocks is a difficult problem. The traditional stress birefringence is basically used in plate tests without cracks or with penetrating cracks. In addition to the 3D-ILC technology, Jv et al. [33] carried out effective research on visualization of the internal stress field by combining 3D printing transparent rock materials with stress freezing and slicing technology.

This method using stress freezing technology to keep the strain unchanged, then one can observe the cut sliced section with stress moire, achieving three-dimensional stress field distributions inside the sample observations. This method cannot be used for real-time monitoring. However, it is still a creative and effective method for the viewing the “black box” of internal cracks in rocks.

In this paper, 3D-ILC combined with stress birefringence technology was used to try to visualize the stress field of the internal crack. It is a different idea. The stress mottling shows high quality and the “petal” distribution at the crack tip indicating that it can reflect the stress distribution characteristics of the internal crack tips. At present, the authors are also carrying out in-depth quantitative theoretical analysis and research and compiling corresponding procedures, and they plan to present thematic results in the future. Here, only qualitative analysis is carried out.

### 3.3. Crack Initiation and Failure Load

Figure 7 shows the characteristic loads of the orifice sample without internal cracks and the orifice sample with internal cracks. It should be noted that the characteristic load of the cracked orifice samples in the figure was the average load of samples with different buried depths.

As can be seen from the figure, the characteristic loads of each sample showed the following rules:

(1) For samples with internal cracks, the proportion of crack initiation load to final failure load was 19.4% for the upper main crack, 22.5% for the lower main crack, and 92.7% for the remote crack. For the sample without internal cracks, the upper main crack was 22.3%, the lower main crack was 22.3%, and the remote crack was 74.6%;
(2) The existence of prefabricated cracks greatly reduced the values of each characteristic loads. Compared with the orifice sample without internal cracks, the average load of the upper main crack, lower main crack, remote crack, and final failure of the specimen with internal cracks were reduced by 41.2%, 31.7%, 15.9%, and 32.3%, respectively.

## 4. Fractography Characteristics

As an independent discipline, “fractography” analyzes the process, type, nature, and mechanism behind material failure by studying the morphology and properties of macro and micro fractography. It can be said that “fractography is a crime scene of mechanics”. Fractography also has many applications in the field of rocks such as scanning electron microscopic (SEM) fracture analysis and fractal rock mechanics. However, in the current research field of transparent rock-like materials, most of the research is focused on the analysis of propagation path and failure modes, and few studies are combined with fractography. In this paper, significant fracture characteristics and mechanisms behind them are presented.

### 4.1. Main Crack Surface Characteristics

#### 4.1.1. Circular Arc Characteristics of Crack Tip

No matter the sample containing the internal crack or not, the crack tip is circular in shape, and its mechanism can be explained in Section 3.1.1.

**Figure 8:**
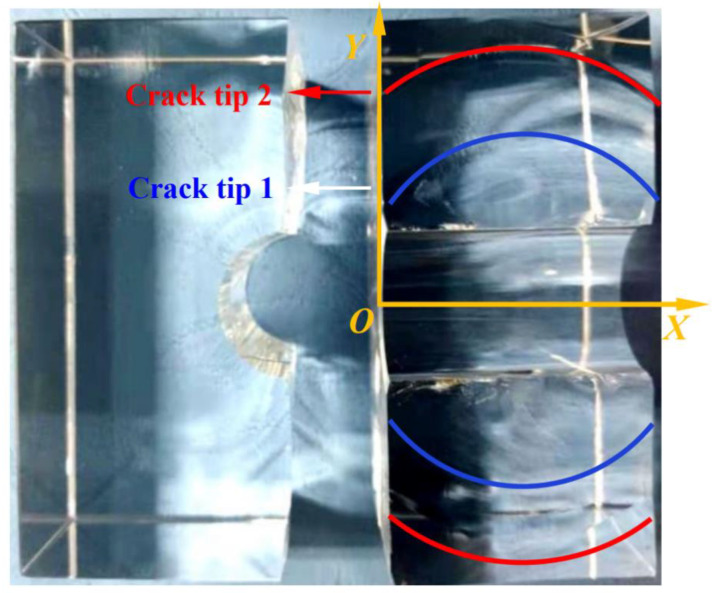
Arc propagation characteristics at crack tip.

As shown in Figure 8, the blue line represents the crack tip when the specimen initiates, and the red line represents the crack tip when the specimen unloads. It can be seen that there were obvious arc-shaped irregular features on the fracture. The rectangular coordinate system, as shown in Figure 8, was defined to fit the arc of the crack surface at the fracture of sample C0-1. The arc equation of crack tip 1 (Equation (9)) and crack tip 2 (Equation (10)):

(9) $${\left(x-20\right)}^{2}+{\left(y-1.81\right)}^{2}=28.54^{2}$$

(10) $${\left(x-20\right)}^{2}+{\left(y-5.99\right)}^{2}=31.82^{2}$$

According to Equations (9) and (10), when the main crack expands, the circular radius of the crack front increases continuously, and the center of the circle is located on the axial center line of the orifice.

#### 4.1.2. Dynamic Fracture Characteristics

**Figure 9:**
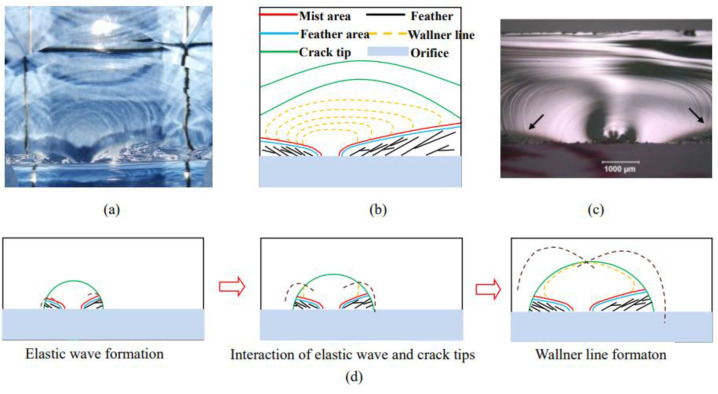
Dynamic crack bifurcation and the characteristic of the Wallner line. (**a**) Main crack fractography; (**b**) Main crack characteristics; (**c**) Wallner line in previous studies; (**d**) Wallner line formation.

Dynamic fracture occurs at the crack initiation moment of the main crack, that is, the “mirror zone”, “atomization zone”, and “feather zone” are presented spreading outward from the crack initiation point as shown in Figure 9a, and the results of uniaxial tensile test in previous experiments [40] are shown in Figure 9c.

Under the action of increasing load, these defects in the solid begin to connect and form cracks and expand outwards. When the crack growth reaches a certain speed, the equilibrium state of static crack growth is broken. At this point, the volume elements in the area near the crack tip inside the sample are affected by non-equilibrium forces, and these volume elements are accelerated to obtain kinetic energy accordingly. The original static solution cannot be established if kinetic energy is ignored, and the static fracture system becomes a dynamic fracture system.

In the dynamic system, the crack growth rate increased continuously until it reached the limit value, and this process showed a radial mirror region, atomization region, and feather region on the fracture surface. These regions also recorded different stages of kinetic energy dissipation: the mirror region was a semicircular plane, the fracture was bright and smooth, and the reflectivity was high, which was formed when the crack propagation rate was low. The atomization zone is the transition zone, which was the semi-ring region outside the mirror area and is the transition zone from slow crack growth to fast crack growth. It is basically in the same plane with the mirror area, and its appearance is characterized by “atomization”; that is, the reflectivity decreases. The reason is that the single smooth crack surface cannot release the kinetic energy of the crack completely under high-speed crack growth, and small-scale fracture occurs on the crack surface, resulting in the increase of surface roughness. The feather area radiated from the atomization area to the surrounding three-dimensional space. The surface of the fracture was very rough when viewed from the plane of the mirror and atomization area. There was still crack propagation outside the plane, and its mechanical mechanism was the dynamic crack bifurcation.

According to the experimental fracture characteristics, this paper analyzed the dynamic crack tip field distortion theory. The state of the near field at the tip of an expanding crack varies with the rate of crack growth. Definition of a “dynamic stress intensity factor”:

(11) $$K^{\prime }=K{\left(v_{t}/v\right)}^{0.5}$$

Then, the angle function, *f_θθ_*(*θ*), has the same form as the static crack tip stress solution:

*f_θθ_*(*θ*) = *σ_θθ_*(2π*r*)^0.5^ (12)

*σ_ij_* = K(2π*r*)^−0.5^*f*_ij_(*θ*) (13)

**Figure 10:**
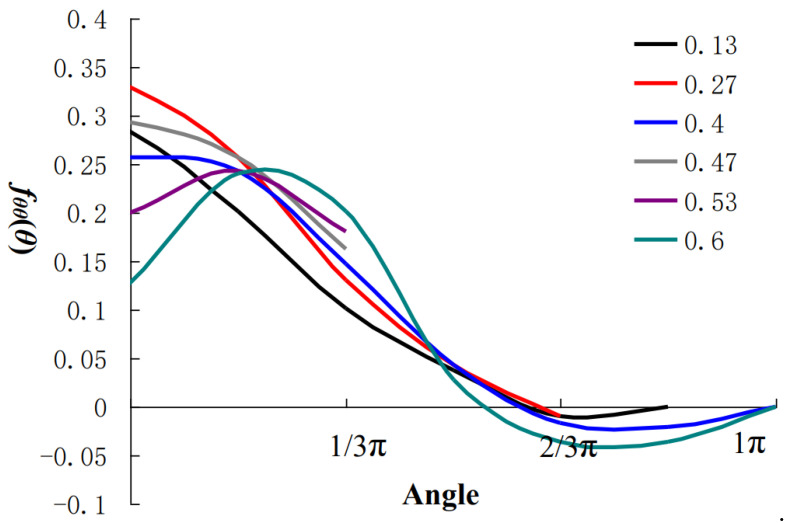
Variation law of angular function.

**Figure 11:**
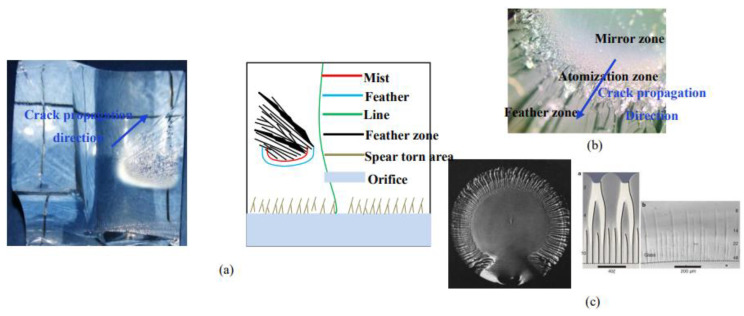
Surface features of remote crack. (**a**) Remote crack surface characteristics; (**b**) Dynamic fracture characteristics; (**c**) Spear torn Characteristics.

Figure 10 shows the component, *σ_θθ_*, calculated for some selected relative crack growth rates, *v*_t_. The instability propagation of the crack occurs when the propagation velocity approaches the theoretical limit rate (the rate of Rayleigh surface waves). As can be seem from Figure 11, when the velocity approached the theoretical limit rate 0.58*v*_t_ (*v*_t_ is the velocity of shear wave propagating in solid), the maximum local tensile stress shifts from 0° on the original crack surface to 25°~35° on the inclined surface. Similarly, the angular distribution of mechanical energy release rate also showed a similar trend. Therefore, in isotropic materials, the crack bifurcation was the inevitable result of the dynamic characteristics of the crack field. 

In this paper, the dynamic fracture characteristic occurred near the orifice area. In the center of the axis of the orifice and the crack, the source can clearly be observed. The dynamic fracture was along the horizontal direction. The authors speculated that when the crack along the axis of the orifice rate of speed and along the main direction of crack propagation rate, it gradually became smaller, presenting the static fracture characteristics.

#### 4.1.3. Secondary Wallner Lines

The Primary Wallner Lines were described in detail in the three-point bending test carried out by the author’s research group [41]. The primary Wallner lines are caused by the elastic stress waves generated by the crack tip during crack propagation, which are reflected at corner points and interact with the expanding crack tip. The propagation direction of the crack tip deviates from the normal path momentarily, and the crack oscillates, leading to the fluctuation of the propagation path of the crack. The reflection of light from these fluctuations makes the crack tip’s interaction with shear waves visible on the surface as continuous arcs. Secondary Wallner lines are caused by the dynamic fracture characteristics of cracks (usually the crack growth reaches its limit rate), and the elastic stress wave generated by the expanded crack front is reflected by the rough surface of the dynamic fracture characteristics of cracks, and finally interacts with the expanded crack front to form, as can be seen in Figure 9d.

### 4.2. Remote Crack Surface Characteristics

#### 4.2.1. Dynamic Fracture Characteristics

The remote crack surface had the same dynamic fracture characteristics as the main crack surface, but the dynamic fracture characteristics of the main crack surface appeared close to the orifice, while the dynamic fracture characteristics of the remote crack surface appeared in the middle of the remote crack surface, and the crack propagation direction was in the mirror zone, atomization zone, and feather zone. The comparison with the literature [28] is shown in Figure 11b.

#### 4.2.2. Characteristics of Tearing III—Shaped Crack

The characteristics of mode III crack in the tearing zone appear at the point where the remote crack is tangent to the orifice. In this paper, combined with the numerical simulation in Section 5, it is judged that this part is mode III crack initiation.

The spear-shaped cracks in the tearing region are closely distributed in line near the orifice. As the cracks continue to extend outwards, 2–3 short lines will converge and merge and continue to extend. The side is stepped, and the linear shape near the orifice is thin, while the outer line shape is thick.

**Figure 12:**
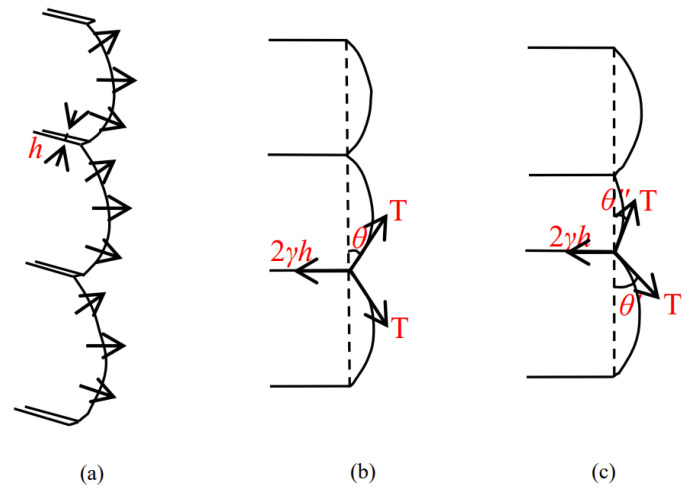
The diagramming of step cracks and the causes of confluence. (**a**) Step crack morphology; (**b**) Force of uniform step; (**c**) Causes of confluence.

Friedel [42] gave a theoretical explanation for the characteristics of “tearing” cracks caused by type III shear dislocation: There is linear tension (*T*) at the front end of the staggered crack. This tension is the unit energy increased by each unit length of the crack front, and it is assumed that *T* is independent of the direction of the crack front and the speed of the crack. The step also has a drag force of 2*γh*, *γ* is the energy required to form a unit surface at the step, and *H* is the height of the step, which is shown in Figure 12. For the step with equal spacing, the crack expands symmetrically with the step as the center, *θ*′ = *θ*″, so that the whole front end of the crack moves forward under the following conditions:

(14) 2γh=2Tsinθ

**Figure 13:**
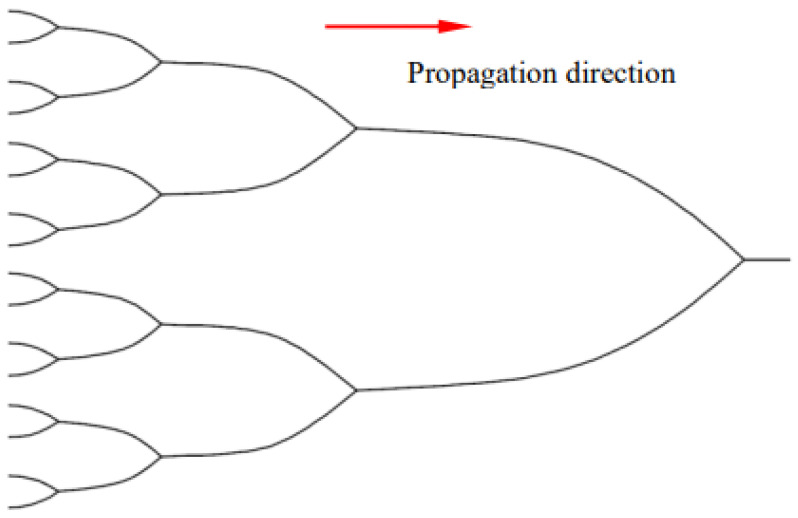
Ideal reverse binary tree pattern.

If the distance between steps is not equal, as shown in Figure 12c, asymmetric forces will appear at the steps, i.e., *θ*′ ≠ *θ*″, thus *T*sin*θ*′ ≠ *T*sin*θ*″. The asymmetric stress will lead to the steps with close spacing being close to each other in the process of crack propagation, and these steps will eventually merge into a new step. As the steps become closer to each other, the *θ*′/*θ*″ ratio increases and the new and old steps merge further, resulting in a “binary tree” shape as shown in Figure 13.

### 4.3. Crack “Penetration”

**Figure 14:**
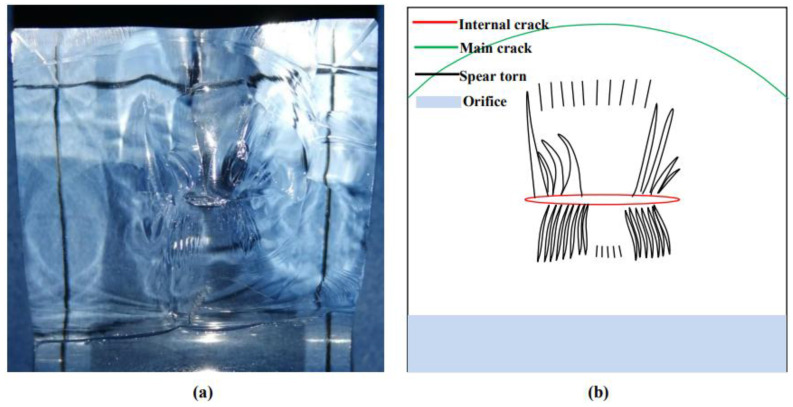
Main crack “penetrating” prefabricated crack. (**a**) Experimental results; (**b**) Schematic diagram.

For the specimen of B2, in the process of main crack propagation, the prefabricated crack would be penetrated. The fractography of the sample is shown in Figure 14.

When the primary crack penetrated the prefabricated crack, it cut off the prefabricated crack and, at the same time, produced mode III spear fracture features on the upper and lower sides of the prefabricated crack (see Section 4.2.1 for its cause). What should be noticed is that the spear crack at the upper part of the prefabricated crack was coarser, while the lower part is thinner, and there is a smaller spear crack extending at the distal end, showing a similar “tentacle shape” shape.

## 5. Numerical Simulations

Three-dimensional crack propagation in fracture mechanics is a difficult problem both theoretically and numerically. At present, there are almost no numerical simulation results involving three-dimensional crack and hole interactions as well as crack penetration under complex stress states. Limited by the existing theoretical and numerical simulation level, qualitative analysis will be carried out in this section, which mainly includes numerical simulation from the following three aspects: (1) the influence of different prefabricated crack burial depths on main crack initiation; (2) study on the propagation process of the main crack; (3) the influence of main crack generation and propagation on remote crack.

### 5.1. Stress Intensity Factor Calculation

The stress intensity factor is calculated by means of m-integral, assuming that the interaction J-integral of the superposition of the two equilibrium states is:

(15) $$\begin{array}{l} \bar{J}=\int_{\Gamma }(\sigma_{ij}^{\left(1\right)}+\sigma_{ij}^{\left(2\right)})\frac{\partial u_{i}^{\left(1\right)}+\partial u_{i}^{\left(2\right)}}{\partial x}(W^{\left(1\right)}+W^{\left(2\right)})\delta_{ij}\frac{\partial q}{\partial x_{j}}ds\\ ={\bar{J}}^{\left(1\right)}+{\bar{J}}^{\left(2\right)}+{\bar{M}}^{\left(1,2\right)} \end{array}$$

For linear elastic materials, strain energy density can be expressed as $W=\sigma_{\mathrm{ij}}\varepsilon_{\mathrm{ij}}/2$. According to Betti reciprocity theorem:

(16) $$W^{{(}^{1,2})}=\sigma_{\mathrm{ij}}{}^{\left(1\right)}\varepsilon_{\mathrm{ij}}{}^{\left(2\right)}=\sigma_{\mathrm{ij}}{}^{\left(2\right)}\varepsilon_{\mathrm{ij}}{}^{\left(1\right)}$$

where:

(17) $${\bar{J}}^{\left(1\right)}=\int_{\Gamma }(\sigma_{ij}^{\left(1\right)}\frac{\partial u_{i}^{\left(1\right)}}{\partial x_{1}}-W^{\left(1\right)}\delta_{ij})\frac{\partial q}{\partial x_{j}}ds$$

(18) $${\bar{J}}^{\left(2\right)}=\int_{\Gamma }(\sigma_{ij}^{\left(2\right)}\frac{\partial u_{i}^{\left(2\right)}}{\partial x_{1}}-W^{\left(2\right)}\delta_{1j})\frac{\partial q}{\partial x_{j}}ds$$

(19) $${\bar{M}}^{\left(1,2\right)}=\left[\begin{array}{c} \sigma_{ij}^{\left(1\right)}\frac{\partial u_{i}^{\left(2\right)}}{\partial x_{1}}+\sigma_{ij}^{\left(2\right)}\frac{\partial u_{i}^{\left(1\right)}}{\partial x_{1}}\\ -\frac{1}{2}(\sigma_{ij}^{\left(1\right)}\varepsilon_{ij}^{\left(2\right)}+\sigma_{ij}^{\left(2\right)}\varepsilon_{ij}^{\left(1\right)})\delta_{1j} \end{array}\right]\frac{\partial q}{\partial x_{j}}ds$$

Using the relationship between energy release rate, *G*, and stress intensity factor, *K*, under two equilibrium states:

(20) $$G=J=\frac{1-\nu^{2}}{E}{\left(K_{I}^{\left(1\right)}+K_{I}^{\left(2\right)}\right)}^{2}+\frac{1-\nu^{2}}{E}{\left(K_{\mathrm{II}}^{\left(1\right)}+K_{\mathrm{II}}^{\left(2\right)}\right)}^{2}+\frac{1+\nu }{E}{\left(K_{\mathrm{III}}^{\left(1\right)}+K_{\mathrm{III}}^{\left(2\right)}\right)}^{2}$$

(21) $$J^{\left(1\right)}=\frac{1-\nu^{2}}{E}{\left(K_{I}^{\left(1\right)}\right)}^{2}+\frac{1-\nu^{2}}{E}{\left(K_{\mathrm{II}}^{\left(1\right)}\right)}^{2}+\frac{1+\nu }{E}{\left(K_{\mathrm{III}}^{\left(1\right)}\right)}^{2}$$

(22) $$J^{\left(2\right)}=\frac{1-\nu^{2}}{E}{\left(K_{I}^{\left(2\right)}\right)}^{2}+\frac{1-\nu^{2}}{E}{\left(K_{\mathrm{II}}^{\left(2\right)}\right)}^{2}+\frac{1+\nu }{E}{\left(K_{\mathrm{III}}^{\left(2\right)}\right)}^{2}$$

(23) $$M^{\left(1,2\right)}=2\left[\frac{1-\nu^{2}}{E}K_{I}^{\left(1\right)}K_{I}^{\left(2\right)}+\frac{1-\nu^{2}}{E}K_{II}^{\left(1\right)}K_{II}^{\left(2\right)}+\frac{1+\nu }{E}K_{III}^{\left(1\right)}K_{III}^{\left(2\right)}\right]ds$$

By selecting a specific auxiliary field, the solution of the stress intensity factors *K*_I_, *K*_II_, and *K*_III_ at the crack tips can be obtained by using the interaction M-integral of the far field through Equation (23).

State 1 is selected as the real state of the problem, state 2 is represented by the asymptotic solution of pure mode I crack:

(24) $$K_{I}^{\left(2\right)}=1,\;K_{\mathrm{II}}^{\left(2\right)}=0,\;K_{\mathrm{III}}^{\left(2\right)}=0$$

We can obtain the equations from Equations (23) and (24):

(25) $$M^{\left(1,2\right)}=2\frac{1-\nu^{2}}{E}K_{I}^{}$$

Therefore:

(26) $$K_{I}=\frac{E}{2(1-\nu^{2})}M^{\left(1,2\right)}=\frac{E}{2(1-\nu^{2})}{\bar{M}}^{\left(1,2\right)}$$

Similarly, state 1 is selected as the real state of the problem, state 2 is represented by the asymptotic solution of pure type II crack, which can then be written as:

(27) $$K_{\mathrm{II}}^{\left(1\right)}=K_{\mathrm{II}}^{},K_{\mathrm{II}}^{\left(2\right)}=1,K_{I}^{\left(2\right)}=0,K_{\mathrm{III}}^{\left(2\right)}=0$$

We can obtain from (16) and (20) that:

(28) $$M^{\left(1,\;2\right)}=2\frac{1-\nu^{2}}{E}K_{\mathrm{II}}^{}$$

(29) $$K_{\mathrm{II}}=\frac{E}{2(1-\nu^{2})}M^{\left(1,2\right)}=\frac{E}{2(1-\nu^{2})}{\bar{M}}^{\left(1,2\right)}$$

Similarly, state 1 is selected as the real state of the problem, state 2 is represented by the asymptotic solution of pure type II crack, and we can obtain:

(30) $$K_{\mathrm{III}}^{\left(1\right)}=K_{\mathrm{III}}^{},K_{\mathrm{III}}^{\left(2\right)}=1,K_{I}^{\left(2\right)}=0,K_{\mathrm{II}}^{\left(2\right)}=0$$

We can then obtain the following equations from Equations (15) and (22):

(31) $$M^{\left(1,2\right)}=2\frac{1+\nu }{E}K_{\mathrm{III}}^{}$$

(32) $$K_{\mathrm{III}}=\frac{E}{2(1+\nu^{})}M^{\left(1,2\right)}=\frac{E}{2(1+\nu^{})}{\bar{M}}^{\left(1,2\right)}$$

Then the stress intensity factor can be obtained from the above equations.

### 5.2. Crack Propagation Criterion

In this paper, the maximum tensile stress criterion (MTS) is adopted as the crack growth criterion. The crack will propagate along the direction of the maximum tensile stress *σ_θ_*_max_, while the circumferential stress is related to mode I stress intensity factor, which can be expressed as:

(33) $$K_{I}{}^{r}(\theta )=\sigma_{\theta \theta }\sqrt{2\pi r}=\cos\frac{\theta }{2}\left[K_{I}\cos^{2}\frac{\theta }{2}-\frac{3}{2}K_{\mathrm{II}}\sin\theta \right]$$

The cracking angle *θ*_0_ can then be obtained:

(34) $$\theta_{0}=\arccos\frac{{3K}^{2}{}_{\mathrm{II}}\pm \sqrt{K^{4}{}_{I}+{8K}^{2}{}_{I}K^{2}{}_{\mathrm{II}}}}{K^{2}{}_{I}+{9K}^{2}{}_{\mathrm{II}}}$$

### 5.3. Numerical Models

**Figure 15:**
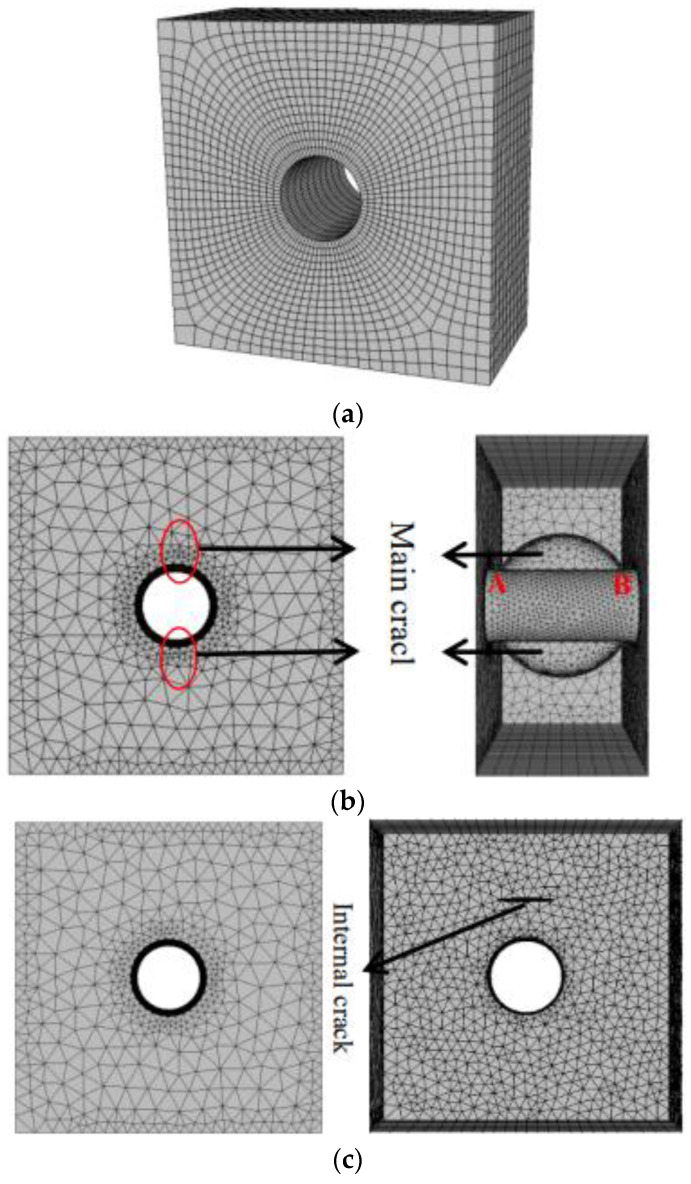
Model mesh division. (**a**) Model 1: Complete orifice model; (**b**) Model 2: Main crack initiation model; (**c**) Model 3: Model with internal cracks. A and B are the start and end point of the crack front.

Three numerical models are established as shown in Figure 15: Model 1: complete orifice model without internal crack; Model 2: the main crack initiation model of the orifice (the size of the main crack is defined according to the experiment results in this paper); Model 3: the model with internal cracks of different buried depths (taking 2 cm as an example). The boundary condition is that the stress boundary is imposed on the upper part of the model and the displacement constraint of fixed three directions is imposed on the lower part of the model.

### 5.4. Numerical Results

#### 5.4.1. Effects of Different Buried Depths on the Initiation of Main Crack

**Figure 16:**
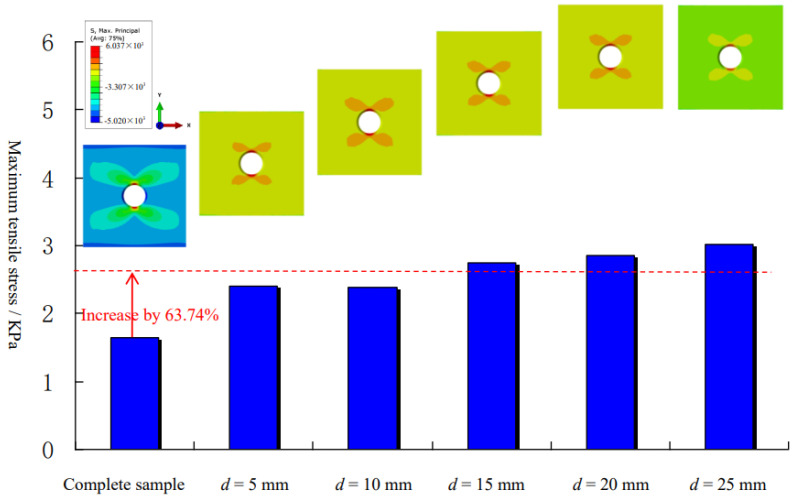
Distribution of maximum tensile stress.

Figure 16 shows the distributions of the maximum tensile stress in Model 1 and Model 3 and the variations of the maximum tensile stress with the prefabricated cracks at different buried depths. The existence of prefabricated cracks greatly changed the stress distributions around the orifice. Under the same load, the maximum tensile stress of the orifice in Model 1 increased by 63.74% on average compared with Model 3. Therefore, the main crack of the orifice sample with internal crack initiates first, which is consistent with the experimental laws. Secondly, by comparing the main cracks at different buried depths in Model 3, it can be found that the greater the buried depth is, the greater the maximum tensile stress around the orifice is. Therefore, in this paper, the main crack penetration phenomenon of group B2 samples is explained as follows: (1) the maximum tensile stress at the orifice of this group of specimens is the largest, and the initiation and propagation of the main crack are more sufficient, so the main crack length is longer; (2) the prefabricated crack is closest to the orifice and is most easily penetrated by the main crack.

#### 5.4.2. Main Crack Propagation Process

**Figure 17:**
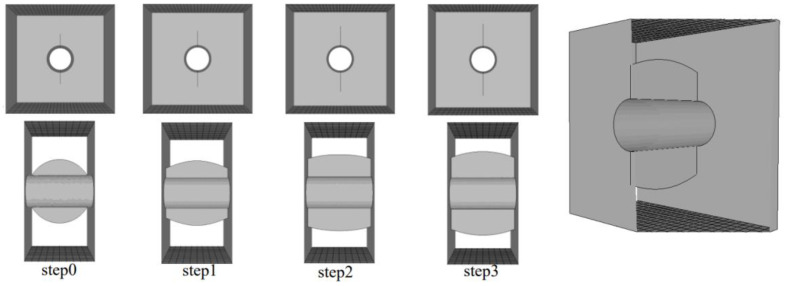
Main crack propagation process.

**Figure 18:**
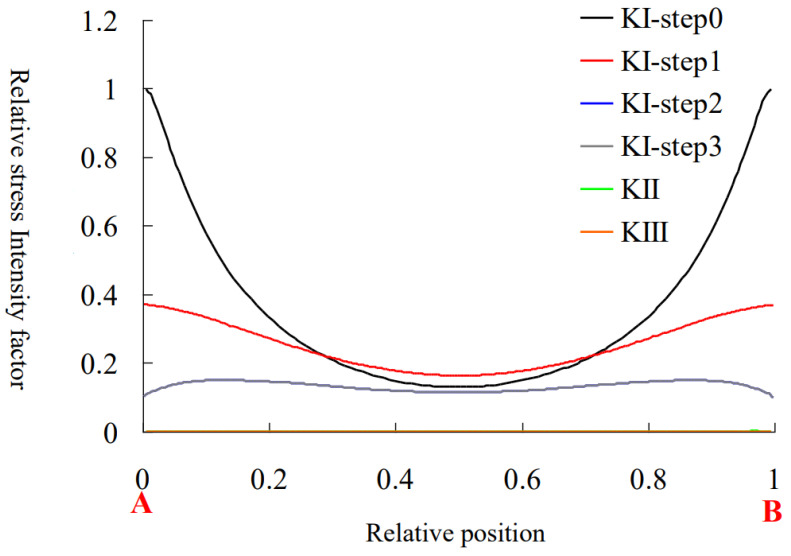
Variation of stress intensity factor at the tip of the main crack. A and B are the start and end point of the crack front.

The main crack propagation process of Model 1 is shown in Figure 17. In order to explain the general rule, the stress intensity factor was normalized, and the relative stress intensity factor was defined as *K*_i_/|*K*_Imax_|. The variation rule of the relative stress intensity factor with the extended time step is shown in Figure 18.

The propagation laws of the main crack can be seen from the figure and listed below:

(1) After initiation, the main crack presents self-similar propagation along the crack surface, which is consistent with the experimental phenomenon. According to the stress intensity factor distributions, the Mode 2 and Mode 3 stress intensity factors of the main crack growth process are 0, which can determine that the main crack is pure mode I fracture;
(2) In the process of main crack propagation, the crack tip presented a circular arc feature. According to the distribution of stress intensity factor, it can be seen that the part near the main crack to the sample surface had a larger *K*_I_, while the stress intensity factor in the middle of the main crack is smaller. Therefore, the part near the sample surface had a faster crack growth, while the middle part had a slower crack growth. Therefore, the radius of the arc gradually increased with the growth of the main crack, which is consistent with the fracture characteristics of the main crack in Section 4.1.1 of this paper;
(3) It can be seen from the pattern of Mode I stress intensity factor over time that the mode I stress intensity factor decreases gradually during the growth of the main crack, which is consistent with the experimental phenomenon that the growth rate of the main crack decreases gradually after initiation in this paper.

#### 5.4.3. Effects of Main Crack Generation and Propagation on Remote Crack

**Figure 19:**
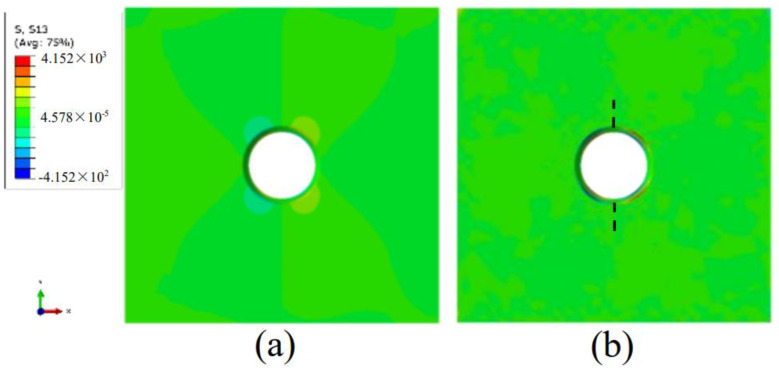
Torsional shear distribution. (**a**) Model 1; (**b**) Model 2.

The distributions of torsional shear stress (S13) between Model 1 and Model 2 are shown in Figure 19. The value of shear stress at the shear concentration of the complete sample (Model 1) (170.1 kpa) was 40.97% of that of the sample with main crack (Model 2) (415.2 kpa). It can be seen that the initiation of the main crack leads to the concentration of the torsional shear stress at 45º, resulting in the initiation of the remote crack tangent to the orifice. Therefore, the remote crack initiation mode was the Mode 3 crack initiation mode, which was also verified by the characteristics of the Mode 3 spear fracture at the Section 4.2 of remote crack tangent to the orifice.

## 6. Conclusions

(1) No matter whether the orifice sample contains internal crack or not, the crack morphology is main crack and remote crack. At the initiation of the main crack, there is a dynamic fracture feature near the orifice, and the tip is arc-shaped. The radius of the arc gradually increases in the process of propagation, while the growth rate becomes slower and slower. When the prefabricated crack buried depth is relatively larger, it “penetrates” the prefabricated crack, and presents a "tentacle-like" shape at the surface of the main crack. The surface of the remote crack is curved and tangential to the orifice. The tangential part presents mode III spear feature, and dynamic fracture occurs on the crack surface;
(2) Stress birefringence can be used to qualitatively monitor the dynamic change of stress in the sample with 3D-ILC internal crack. The existence of internal crack changes the original “flame” moire distribution of the orifice sample, and the moire at the prefabricated crack tip presents "petal" characteristics;
(3) The presence of prefabricated cracks reduced the characteristic load of the sample, the upper main crack decreased 41.2%, the lower main crack decreased 31.7%, the remote crack decreased 15.9%, and the failure load decreased 32.3%;
(4) The results of qualitative stress analysis of the orifice sample were consistent with the crack initiation laws of the main crack and remote crack, and the numerical simulation of the crack propagation process based on the K distributions of M integral and MTS was consistent with the main crack propagation laws;
(5) 3D-ILC can be applied to the study of crack propagation in brittle solids. Compared with the current mainstream method of transparent rock research, the embedded casting method has certain progress in approaching rock brittleness, crack authenticity, stress field visualization, fracture characteristics and other aspects.

## Figures and Tables

**Table 1:** Research scheme.

Samples	Schematic Diagram	Number	Buried Depths/cm	Whether to Crush	Purpose
Samples without internal cracks	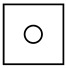	A0	/	Yes	Observe crack propagation
		C0-1		No	Observe main cracks
		C0-2		No	Observe remote cracks
Samples with internal cracks	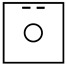	B0.5	0.5	Yes	Observe crack propagation
		C0.5-1	0.5	No	Observe main cracks
		C0.5-2	0.5	No	Observe remote cracks
	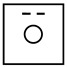	B1	1	Yes	Observe crack propagation
		C1-1	1	No	Observe main cracks
		C1-2	1	No	Observe remote cracks
	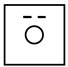	B1.5	1.5	Yes	Observe crack propagation
		C1.5-1	1.5	No	Observe main cracks
		C1.5-2	1.5	No	Observe remote cracks
	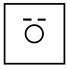	B2	2	Yes	Observe crack propagation
		C2-1	2	No	Observe main cracks
		C2-2	2	No	Observe remote cracks

## Data Availability

All the data, models, or codes that support the findings of this study are available from the corresponding author upon request.
